# Supplementary material for: SARS-CoV-2 rapidly evolves lineage-specific phenotypic differences when passaged repeatedly in immune-naïve mice
Source: Commun Biol. 2024 Feb 16;7:191. doi: 10.1038/s42003-024-05878-3 (PMC10873417; doi:10.1038/s42003-024-05878-3)
Supplement: Supplementary file 2 — Supplementary material [file 42003_2024_5878_MOESM2_ESM.pdf]

Supplementary Table 1

Any variant that clearly changed in MAF during the study

Variant	Protein Change	Annotation (* means druggable for human interacting protein)	Potentially Interacting human proteins (* indicates reported as druggable)	Proteins presently implicated with COVID-19
A929G	ORF1ab I222V, nsp2	Protein interacting*	GIGYF2, **FKBP15***, WASHC4, RAP1GDS1, POR, **eIF4E2***, SLC27A2	GIGYF2 (PMID 35878012)
C3042T	ORF1ab P926L	None	None	None
C4763T	ORF1ab H1500Y	None	None	None
C10341T	ORF1ab P3359L, nsp5	Protein interacting*	**HDAC2***	**HDAC2** (PMID 36758802)
G10533T	ORF1ab C3423F, nsp5	Protein interacting*	**HDAC2***	**HDAC2** (PMID 36758802)
C10809T	ORF1ab P3515L, nsp5	Protein interacting*	**HDAC2***	**HDAC2** (PMID 36758802)
A12555C	ORF1ab E4097A	Protein interacting	MPHOSPH10, SRP72, ATE1, NSD2, SRP19, SRP54, MRPS25, MRPS5, EXOSC2, LARP7, MEPCE, MRPS2, HECTD1, AATF, EXOSC3, EXOSC5, SEPSECS, CCDC86, NOL10, NARS2, EXOSC8, MRPS27, DDX10, NGDN	MPHOSPH10 (PMID 35045126), EXOSC2 (PMID 36241425), LARP7 (PMID 33647885), MEPCE (PMID 33883027), MRPS2 (PMID 36371196)
A12884G	ORF1ab T4207A	Protein interacting*	**NUP214***, FBN2, FBN1, GTF2F2, DCAF7, EIF4H, **NUP54***, MIB1, SPART, FBLN5, **NUP62***, MAT2B, **NUP58***, **NUP88***, ZNF503, **NEK9***	MAT2B (PMID 36371196)
C21557T	Noncoding	None	None	None
A21651C	Spike N30T	Protein interacting, vaccine target, mutation may confer drug resistance	GOLGA7, ZDHHC5	GOLGA7 (PMID 34961524), ZDHHC5 (PMID 34961524)
C22674T	Spike S371F	Protein interacting, antibody escape, vaccine target, Omicron VOC	GOLGA7, ZDHHC5	GOLGA7 (PMID 34961524), ZDHHC5 (PMID 34961524)
G23593T	Spike Q677H	Protein interacting, vaccine target	GOLGA7, ZDHHC5	GOLGA7 (PMID 34961524), ZDHHC5 (PMID 34961524)
C23606T	Spike R682W	Protein interacting, vaccine target, site of positive selection	GOLGA7, ZDHHC5	GOLGA7 (PMID 34961524), ZDHHC5 (PMID 34961524)
C23613T	Spike A684V	Protein interacting, vaccine target	GOLGA7, ZDHHC5	GOLGA7 (PMID 34961524), ZDHHC5 (PMID 34961524)
C24237T	Spike A892V	Protein interacting, vaccine target, potential pathogenic insertion/deletion	GOLGA7, ZDHHC5	GOLGA7 (PMID 34961524), ZDHHC5 (PMID 34961524)
A25107G	Spike E1182G	Protein interacting, vaccine target	GOLGA7, ZDHHC5	GOLGA7 (PMID 34961524), ZDHHC5 (PMID 34961524)
TAATC26160TATC	ORF3a NP257-258IX	Protein interacting, site of positive selection	HMOX1, TRIM59, ARL6IP6, VPS39, CLCC1, VPS11, SUN2, ALG5	TRIM59 (PMID 36523164)
T27638C	ORF7a V82A	Protein interacting, Delta VOC	HEATR3, MDN1	None
C27752T	ORF7a T120I	Protein interacting, Delta VOC	HEATR3, MDN1	None
CA27752TC	ORF7a T120I	Protein interacting, Delta VOC	HEATR3, MDN1	None
C27925T	ORF8 T11I	None	None	None
G28237T	ORF8 R115L	Protein interacting*	COL6A1, PCSK6, **LOX***, **DNMT1***, NPC2, CISD3, ITGB1, PLAT, STC2, TOR1A, **PLOD2***, INHBE, CHPF2, UGGT2, FBXL12, PLEKHF2, SMOC1, POFUT1, **FKBP10***, ERLEC1, IL17RA, ADAMTS1, HS6ST2, SDF2, NEU1, GDF15, TM2D3, SIL1, EDEM3, ERP44, PVR, NGLY1, OS9, ADAM9, NPTX1, POGLUT2, POGLUT3, ERO1B, PLD3, FOXRED2, CHPF, PUSL1, HYOU1, MFGE8, FKBP7, GGH, EMC1	COL6A1 (PMID 35468151), **LOX** (PMID 34616409), PLAT (PMID 34786557), **PLOD2** (PMID 33328453), FBXL12 (PMID 34183789), **FKBP10** (PMID 35571107), IL17RA (PMID 33723527), HS6ST2 (PMID 32970989), NEU1 (PMID 36714013), GDF15 (PMID 35251002), ADAM9 (PMID 34698500), PLD3 (PMID 36182629)
C28320T	Nucleoprotein T16M	Protein interacting*	PABPC1, RPL36, RRP9, G3BP2, RBM28, DDX21, MOV10, UPF1, SNIP1, FAM98A, **LARP1***, PABPC4, G3BP1, **CSNK2B***, **CSNK2A2***, CHMP2A, SLC9A3R1, CSDE1, TOMM70, **MARK3***, **MARK2***, **DCTPP1***, MARK1, PTBP2, BAG5, DPH5	DDX21 (PMID 35107372), MARK1 (PMID 36327219)
G28368A	Nucleoprotein R32H	Protein interacting*	PABPC1, RPL36, RRP9, G3BP2, RBM28, DDX21, MOV10, UPF1, SNIP1, FAM98A, **LARP1***, PABPC4, G3BP1, **CSNK2B***, **CSNK2A2***, CHMP2A, SLC9A3R1, CSDE1, TOMM70, **MARK3***, **MARK2***, **DCTPP1***, MARK1, PTBP2, BAG5, DPH5	DDX21 (PMID 35107372), MARK1 (PMID 36327219)
TACGA29748TA	Noncoding	None	None	None
T29821G	Noncoding	None	None	None

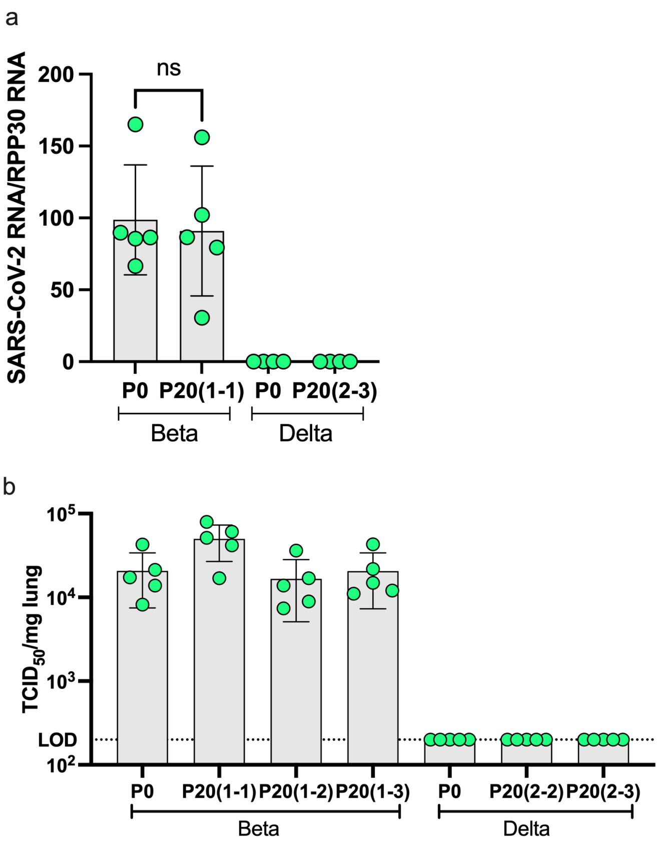

Supplementary Figure 1. Infection of C57BL6 mice with P0 and P20 Beta and Delta viruses. Mice (n = 5/group) were infected intranasally with 3000 $TCID_{50}$ of P0 and P20 Beta (1-1, 1-2 or 1-3) and Delta (2-2 or 2-3) viruses. On the third day post infection, mice were euthanized and lungs collected for RNA extraction and infectious viral load determination. a) Mean +/- SD of normalized SARS-CoV-2 RNA copies as determined using ddPCR. b) Mean $TCID_{50}$/mg of lung tissue. LOD=limit of detection. ns signifies not significant, using a two-tailed paired t-test.

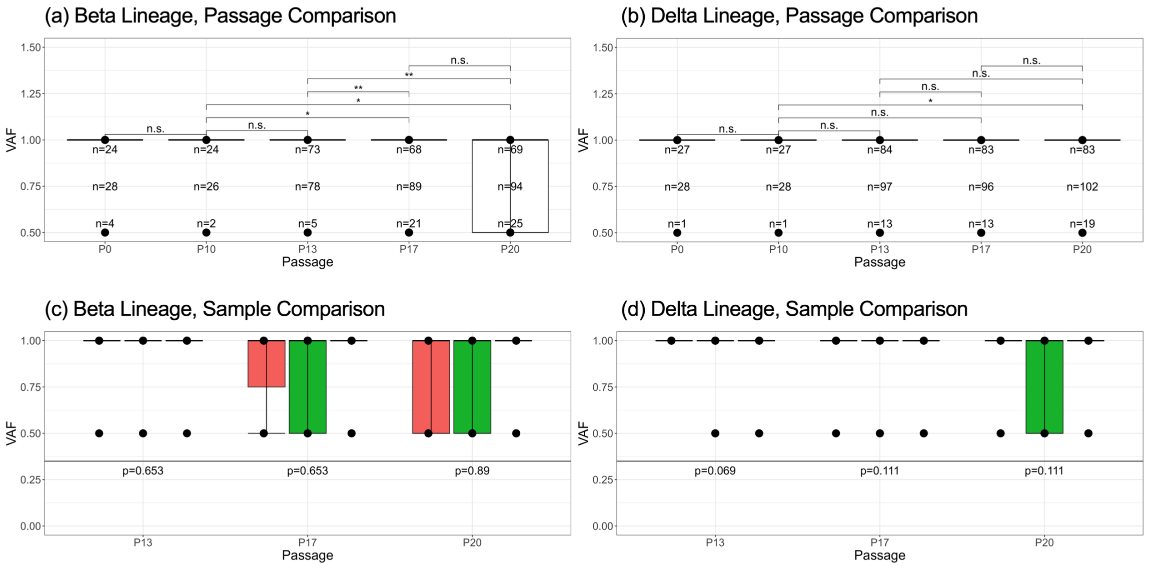

Supplemental Figure 2. Boxplots of variant allele frequency by (a-b) passage and (c-d) sample. Each N refers to the number of variant alleles for each variant allele frequency (VAF), determined using freebayes, and in total against the NC_045512.2 reference sequence. Single samples were sequenced for P0 and P10 with three samples from P13, P17, and P20. Box fill in (c-d) signifies sample number. For significance, n.s.: adjusted p-value > 0.05, *: adjusted p-value > 0.01, **: adjusted p-value ≤ 0.01, using two-tailed unpaired t-tests.

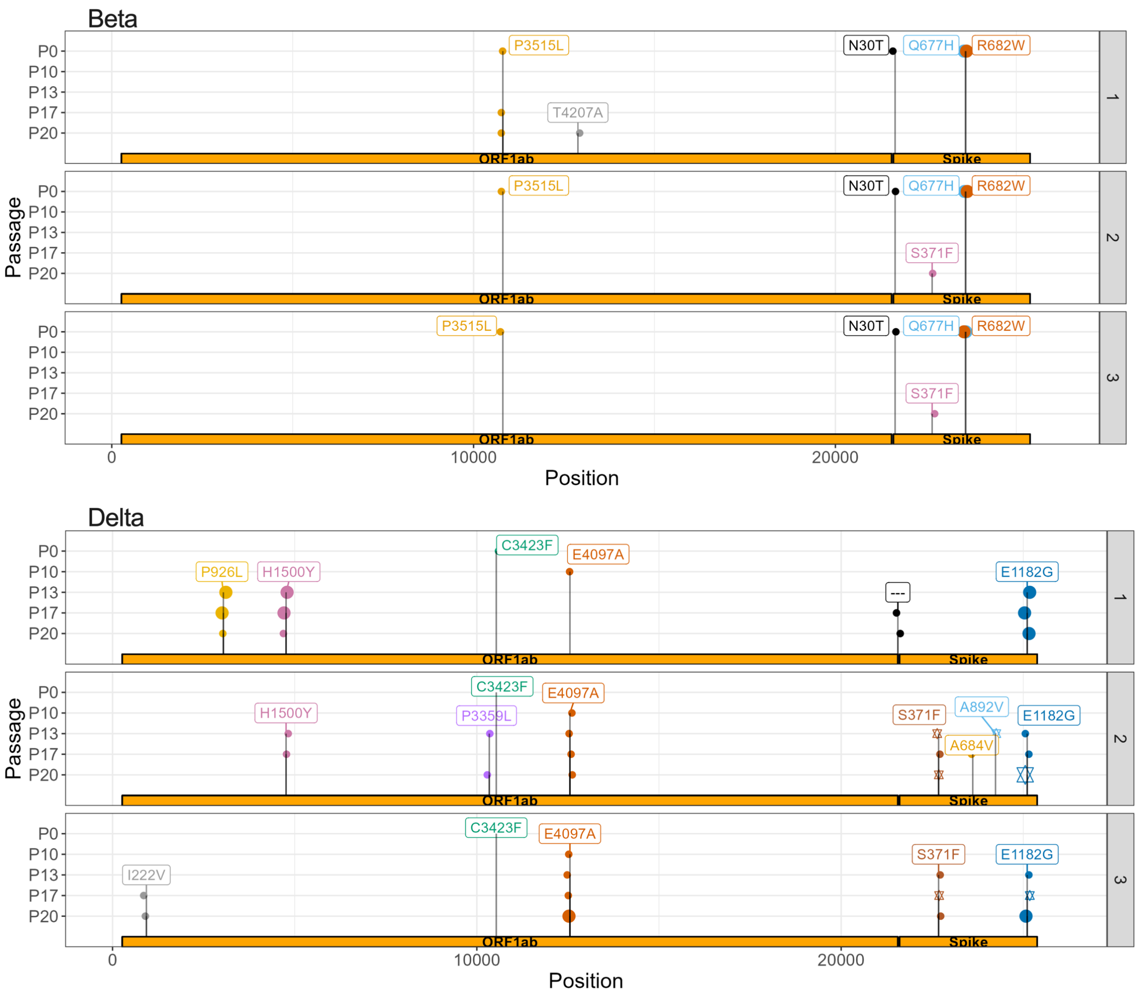

Supplemental Figure 3. More novel, high confidence, non-synonymous, changing alleles appeared in Delta versus Beta in the ORF1ab coding region. VAF: variant allele frequency. P refers to passage. Variant allele incidence by sample in Delta lineage virus. Larger points represent variant alleles with a within-sample, Illumina-derived allele-frequency of 1.0, smaller ones 0.5. Stars in a given passage represent alleles that are likely co-inherited per Nanopore sequencing.
