# Supplementary material for: SARS-CoV-2 rapidly evolves lineage-specific phenotypic differences when passaged repeatedly in immune-naïve mice
Source: Commun Biol. 2024 Feb 16;7:191. doi: 10.1038/s42003-024-05878-3 (PMC10873417; doi:10.1038/s42003-024-05878-3)
Supplement: Supplementary file 3 — Description of Additional Supplementary Files [file 42003_2024_5878_MOESM3_ESM.pdf]

Description of Additional Supplementary Files

File name: Supplementary Data 1

Description: Numerical source data for the graphs in Figures 2-8.
